# Enhanced Neonatal Brain Responses To Sung Streams Predict Vocabulary Outcomes By Age 18 Months

**DOI:** 10.1038/s41598-017-12798-2

**Published:** 2017-09-29

**Authors:** Clément François, Maria Teixidó, Sylvain Takerkart, Thaïs Agut, Laura Bosch, Antoni Rodriguez-Fornells

**Affiliations:** 1 Cognition and Brain Plasticity Group, Bellvitge Biomedical Research Institute IDIBELL, L’Hospitalet de Llobregat, Barcelona, Spain; 2 Department of Cognition, Development and Educational Psychology, University of Barcelona, Barcelona, Spain; 3 Institut de Recerca Pediàtrica Hospital Sant Joan de Déu, Barcelona, Spain; 4 Aix Marseille Univ, CNRS, INT, Inst Neurosci Timone, Marseille, France; 5 Department of Neonatalogy, Hospital Sant Joan de Déu, Barcelona, Spain; 6 Institut de Neurociències, University of Barcelona, Barcelona, Spain; 7 Institució Catalana de Recerca i Estudis Avançats, ICREA, Barcelona, Spain

## Abstract

Words and melodies are some of the basic elements infants are able to extract early in life from the auditory input. Whether melodic cues contained in songs can facilitate word-form extraction immediately after birth remained unexplored. Here, we provided converging neural and computational evidence of the early benefit of melodies for language acquisition. Twenty-eight neonates were tested on their ability to extract word-forms from continuous flows of sung and spoken syllabic sequences. We found different brain dynamics for sung and spoken streams and observed successful detection of word-form violations in the sung condition only. Furthermore, neonatal brain responses for sung streams predicted expressive vocabulary at 18 months as demonstrated by multiple regression and cross-validation analyses. These findings suggest that early neural individual differences in prosodic speech processing might be a good indicator of later language outcomes and could be considered as a relevant factor in the development of infants’ language skills.

## Introduction

Very early in life human neonates already exhibit remarkable auditory learning capacities^1,2^. The ability to detect pitch changes is already functional prenatally as early as after 27 weeks of gestation^3^ and prenatal exposure to musical pieces induces memory traces that are still present 4 months after birth^4^. The early ability to process auditory stimuli during the last three months of gestation is associated to the fast migration of neurons from the ventricular zone to the cortical plate which may already determine the specific columnar organization of sensory areas observed in the left and right auditory cortices^5,6^. Interestingly, while human newborns already activate a left-lateralized fronto-temporal network including cortical perisylvian regions (superior temporal, inferior frontal and inferior parietal cortices) in response to linguistic stimuli^7–9^, a right lateralized activation is observed for voice or music processing^10,11^. The different patterns of activations observed for specific features of the auditory input may well be explained by differences in temporal sensitivity of the left and right auditory cortices^12,13^. Interestingly, two-day-old newborns can also build up temporal expectations and pick up the rhythmical regularities in the auditory input^14^. Furthermore, newborns show sensitivity to the rhythmic properties of speech and music^15,16^ and their cry melodies have been found to be already language specific^17^. In this context, parents’ use of infant-directed speech (IDS) containing exaggerated prosodic properties has been found to be important in facilitating speech perception and language acquisition^18–20^.

One of the earliest problems infants face in language learning is breaking the continuous speech input into word-form units, which is a necessary step in language acquisition. As opposed to written language, speech unfolds in time and no systematic cues signal word boundaries. One of the possible mechanisms proposed to solve this segmentation problem relies on humans’ ability to track the statistical regularities present in the speech signal, based on the computation of transitional probabilities between adjacent syllables^21^. Using behavioural measures, seven- to nine-month-old infants have been shown to use statistical information to extract word-form units from speech streams^22^. Recent work has also revealed newborns’ successful statistical learning in the visual, auditory and speech domains^23–25^.

Prosodic cues such as lexical stress or pauses have also been shown to be crucial for successful word segmentation in both adults^26,27^ and infants^22,28^. Interestingly, a recent EEG study has provided the first experimental evidence showing that IDS may facilitate statistical learning in sleeping neonates. This facilitation was reflected by enhanced brain responses for IDS as compared to adult-directed speech (ADS) material and thus confirmed the benefit of prosodic cues on early word-form segmentation^29^.

Following this idea, we investigated the benefit of prosodic cues on word-form segmentation in newborns by directly comparing brain activity elicited by flat or melodically enriched syllable sequences of connected speech of an artificial language in a within-subject design. With this aim, we developed an innovative procedure in which following an initial learning phase, newborns were exposed to an implicit test phase in which statistically illegal word-forms were pseudo-randomly inserted. Crucially, the brain activity collected in our paradigm allowed us to study both learning-related electrophysiological modulations (learning phase) and the outcome of the learning process (implicit test phase). The presence of specific neural signatures associated to the detection of structural violations (i.e. illegal word-forms) may indirectly reflect the strength of the underlying word-form memory traces created during the preceding learning phase^30,31^.

**Figure 1:**
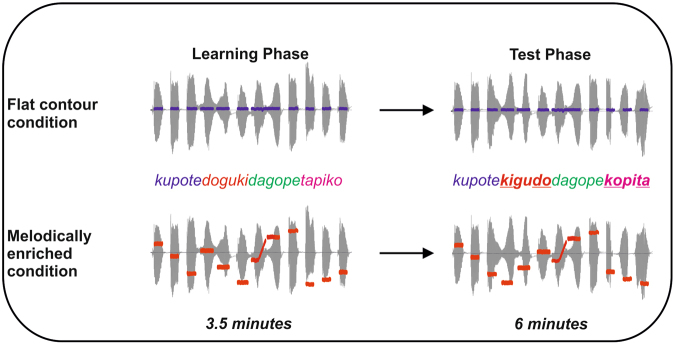
After a learning phase of 3.5 minutes (left side, learning phase), an implicit test (right side, test phase) was performed in which illegal word-forms violating the statistical structure (e.g. a legal ABC word-form in the language would become a CBA illegal word-form) appeared at pseudo-random positions in the stream. In the flat contour condition, all the syllables were spoken on the same pitch, thus resulting in a monotonous stream of syllables in which the only cue to word segmentation were the transitional probabilities between adjacent syllables. In the melodically enriched condition, each syllable was associated to a unique pitch, thus resulting in a continuous stream of sung syllables in which transitional probabilities and pitch modulations could be used for word segmentation. The blue traces represent the constant pitch used in the flat contour condition for both learning and test phases. The red traces represent the varying pitches used in the melodically enriched condition for both learning and test phases. In both conditions, the syllable duration was set to 350 ms thus leading to 1050 ms tri-syllabic pseudo-words.

We measured event-related brain potentials (ERPs) in 28 healthy 2- to 4-day-old sleeping human neonates exposed to continuous speech streams composed of four tri-syllabic items with and without pitch contour modulations (“flat contour condition” and “melodically enriched condition”; see Fig. 1, for experimental design). Considering recent data obtained with IDS material^29^, our working hypothesis was that melodic cues consistently coupled with statistical information in the sung streams would enhance speech segmentation in neonates. This benefit should be reflected during the learning phase by different dynamics of the ERP components elicited by melodically enriched and flat contour speech streams. Considering previous studies reporting the modulation of either a fronto-central positivity or a fronto-central negative ERP component in the 200–500 ms latency band post word-onset during speech segmentation tasks both in adults^30–33^ and neonates^25,29^, we focused our analyses in the ERP components that developed during this time-window. For the analyses of the implicit test phases, we capitalized on previous research showing that neonates can detect low level^34^ as well as more complex/abstract changes in the auditory inputs^35^. These studies have consistently shown that deviant stimuli rarely occurring in a sequence of standard stimuli elicited a mismatch response (MMR), considered as an electrophysiological index of pre-attentive, implicit auditory processing and may reflect the formation of an echoic memory trace within the auditory cortex^36^. Here, we included illegal items containing two syllabic order changes, one in the first and the other in the last syllable position. Considering previous studies using similar types of deviants^30,31^, we expected to observe enhanced neural signatures of illegal word-form detection, indexed by the presence of a MMR elicited by the first and second syllabic order changes, developing around 150 ms after syllable onset, in the melodically enriched condition when compared to the flat contour condition, indexing more efficient word-form extraction.

Interestingly, previous studies have demonstrated a link between behavioural and electrophysiological indices of early speech discrimination abilities for native and non-native phonemes and later language outcomes (expressive and receptive vocabulary)^37^. In addition, previous research on early word segmentation skills using natural speech stimuli have also shown that behavioural and electrophysiological data obtained during speech segmentation tasks in infants ranging from 7.5 to 12 months of age predicted language outcomes later in development^38–40^. Based on these previous studies, we aimed to provide first evidence for the predictive power of neonatal EEG signatures of speech segmentation for later language outcomes, assessed from vocabulary measures at 18 months of age. With this aim, we longitudinally followed the neonates enrolled in the EEG study and gathered measures of expressive vocabulary with the MacArthur Bates Developmental Inventory (MCDI) as well as the language subscale scores from the Bayley Scales of Infant Development (BSID-III) at 18 months of age. In order to explore the link between EEG signatures of early speech segmentation and expressive vocabulary at 18 months, we used Pearson correlations, linear multiple regression and cross-validation procedures with the EEG features reflecting the learning brain dynamics (for both flat contour and melodically enriched conditions), as well as the significant MMR found during the test phases of the melodically enriched condition, as predictive indices. Specifically, we hypothesized that neonatal brain sensitivity to melodically enriched speech streams could be predictive of expressive vocabulary at 18 months of age.

## Results

### Learning phases

**Figure 2:**
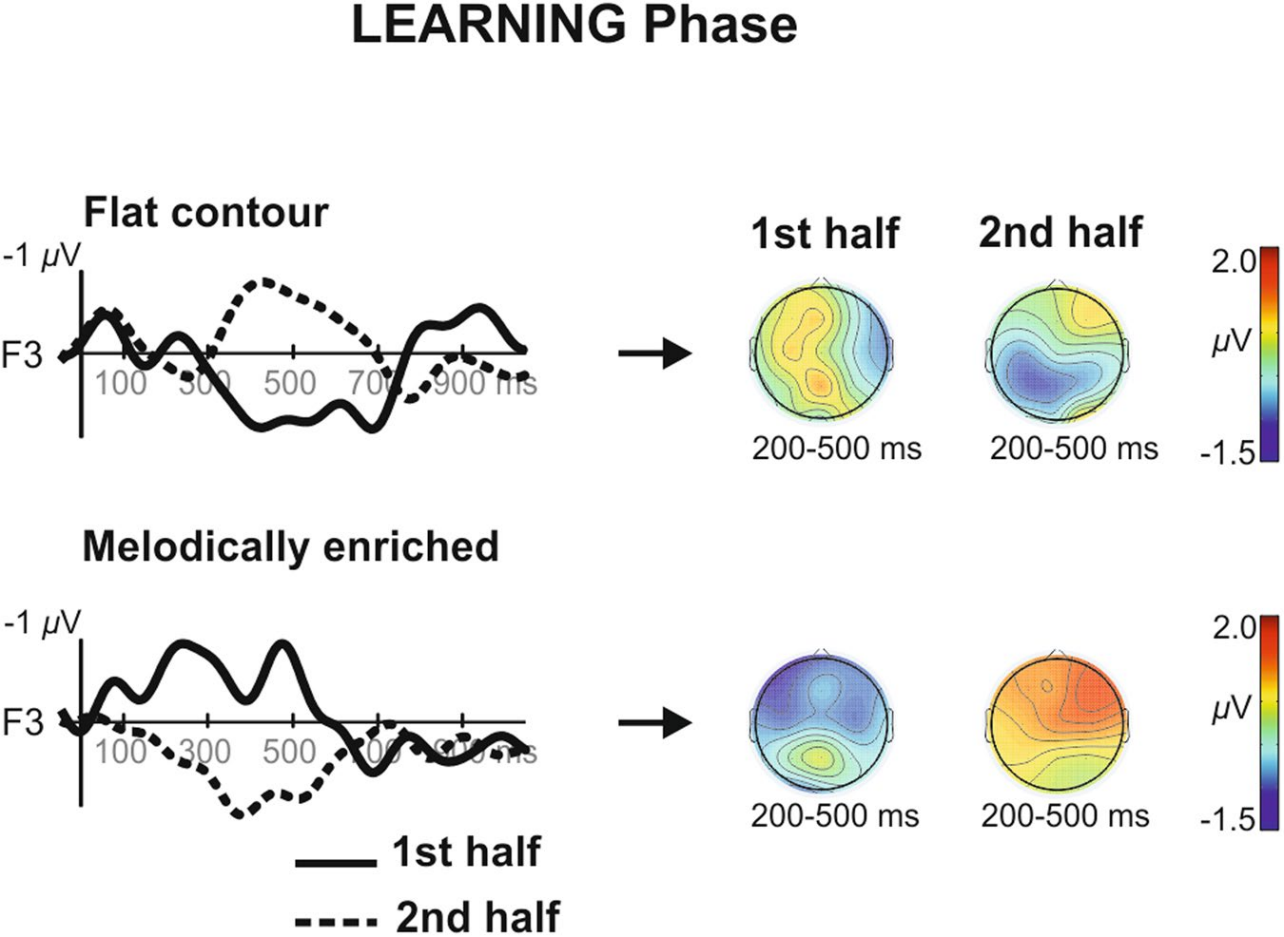
Time-course of the Learning effects. Grand averages ERPs at F3 electrode across 27 newborns recorded during each block (thick = 1^st^ block, dotted = 2^nd^ block) of the learning phases in both conditions (flat and melodically enriched). The isovoltage topographical maps show the distribution of the mean amplitude in the 200–500 ms latency band for each learning block.

The EEG data from the learning phases were divided in two non-overlapping blocks for both conditions. This was done in order to track the dynamic evolution of the ERPs through exposure to the streams (see Fig. 2 and Supplementary Fig. 1). The results of the repeated-measures analysis of variance (ANOVA) with condition (flat contour and melodically enriched), blocks (block 1 and block 2) and electrode (16 levels) as within-subjects factors showed different patterns of ERP modulation as a function of exposure as revealed by a significant condition by block interaction (*F*(1,25) = 5.02; *P* = 0.03; ƞ_p_^2^ = 0.17). In the flat contour condition, a clear broadly distributed positivity was observed in the first block while a central-parietal negativity was found during the second block. Nonetheless, the between blocks pairwise comparison performed on the mean amplitude across all electrodes showed no significant difference between blocks (1^st^ block: 0.35 μV +/− 1.41; 2^nd^ block: −0.11 μV +/− 1.72, post-hoc test: *P* = 0.32). By contrast, the melodically enriched condition elicited a fronto-central negative ERP component during the first block (−0.48 μV +/− 1.65) whereas a clear positivity was observed in the second block (0.88 μV +/− 2.36). The between blocks post-hoc LSD tests performed on the mean amplitude across all electrodes in the melodically enriched condition showed significant differences (Post-hoc LSD test: *P* = 0.03). The interaction condition by block by electrode was not significant, nor was the main effect of block or condition (all *P*’s > 0.3). Despite visual differences in the scalp distribution of the negative components found in the different learning blocks (see isovoltage maps at Fig. 2, right side), a further comparison (repeated-measures ANOVA with the factors condition and electrodes) between the topography of both components showed no significant differences (main effect of condition *F*(1,25) = 0.60; *P = *0.44; ƞ_p_^2^ = 0.02; condition by electrode interaction *F*(1,25) = 2.06; *P = *0.11; ƞ_p_^2^ = 0.74).

### Test phases

**Figure 3:**
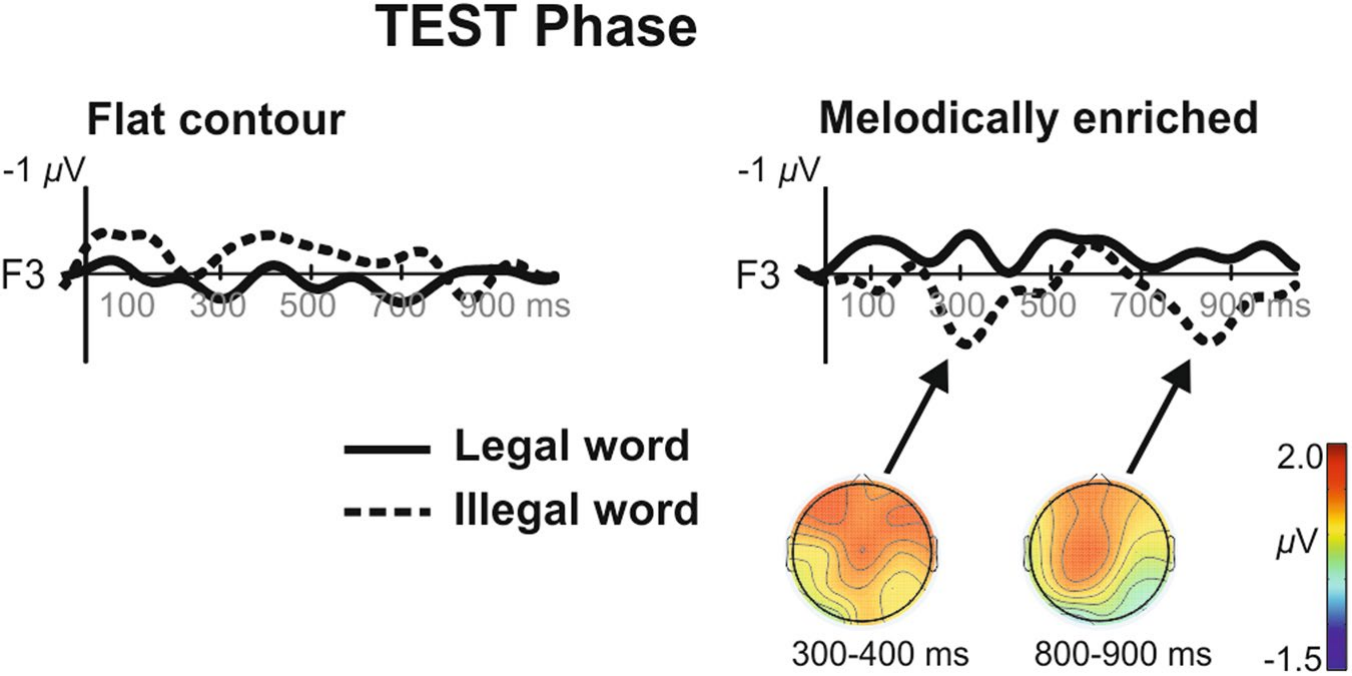
Grand averages ERP at F3 electrode across 26 newborns for legal and illegal word (thick = legal, dotted = illegal words) of the test phases in both conditions. The topographical maps show the distribution of the mean amplitude for illegal words in the significant time-windows.

During the test phases, and as a first step that aimed to detect the presence of a MMR in each condition, we compared the mean amplitude of the difference waveform (illegal minus legal words) against zero separately for each condition (see Table 1). Because during the test phases, the illegal words which were built by reversing the order of the first and last syllables composing each of the words (see Fig. 1), we expected to observe two MMR developing after the first and last syllables (see ERP results in Fig. 3, and Supplementary Fig. 2). Results of this analysis revealed no significant differences in the flat contour condition in neither of the electrodes and time-windows. By contrast, we found significant differences against zero in the melodically enriched condition in two time-windows (300–400 and 800–900 ms) with even more significant effects for the second time-window (see Table 1).

**Table 1:** Test phase: Results of the *t*-tests comparing the difference waveform (illegal minus legal words) against zero for the two time-windows (300–400 and 800–900 ms) and for the 10 electrodes in the two conditions.

Flat Contour	300–400						800–900					
	M	SD	*t*	*P*	*P*_*FDR*_	Cohen’s d	M	SD	*t*	*P*	*P*_*FDR*_	Cohen’s d
Fp1	−0.07	2.1	−0.2	0.87	0.94	0.02	−0.13	1.4	−0.5	0.64	0.88	0.19
Fp2	−0.19	2.2	−0.4	0.66	0.94	0.27	−0.24	1.1	−1.0	0.31	0.88	0.41
F4	0.06	1.9	0.1	0.88	0.94	0.19	−0.08	1.3	−0.3	0.76	0.88	0.12
Fz	0.03	1.8	0.1	0.94	0.94	0.08	−0.07	1.5	−0.2	0.80	0.88	0.09
F3	−0.38	2.3	−0.8	0.42	0.94	0.37	−0.17	1.2	−0.7	0.50	0.88	0.32
T7	−0,98	2.1	−2.3	0.02	0.20	0.8	−0.47	1.3	−1.9	0.07	0.70	0.76
C3	−0.34	2.6	−0.7	0.52	0.94	0.21	−0.21	1.7	−0.6	0.52	0.88	0.26
Cz	−0.21	3.1	−0.3	0.74	0.94	0.31	−0.05	1.8	−0.1	0.89	0.89	0.06
C4	−0.04	2.3	−0.1	0.94	0.94	0.06	0.12	1.5	0.4	0.67	0.88	0.17
T8	0.66	2.2	1.6	0.13	0.65	0.05	0.09	1.1	0.4	0.70	0.88	0.15
**Melodically Enriched**	**300–400**						**800–900**					
	**M**	**SD**	***t***	***P***	***P***_***FDR***_	**Cohen’s d**	**M**	**SD**	***t***	***P***	***P***_***FDR***_	**Cohen’s d**
Fp1	1.27	2.3	2.9	0.008	0.07	1.1	1.49	2.0	3.8	**0.0008**	0.003	**1.5**
Fp2	1.33	2.8	2.4	0.02	0.07	0.9	1.53	2.0	3.9	**0.0006**	0.003	**1.5**
F4	1.13	2.9	1.9	0.06	0.10	0.8	1.17	1.9	3.1	**0.004**	0.01	**1.2**
Fz	1.06	3.2	1.7	0.10	0.14	0.7	1.15	2.1	2.8	**0.009**	0.01	**1.1**
F3	1.29	3.0	2.2	0.03	0.07	0.9	1.32	2.3	2.9	**0.007**	0.01	**1.2**
T7	0.77	0.5	1.6	0.12	0.15	0.6	0.48	1.9	1.3	0.2	0.20	0.5
C3	1.10	2.7	2.1	0.04	0.08	0.8	1.02	2.0	2.6	**0.01**	0.01	**1.0**
Cz	1.40	3.2	2.2	0.03	0.07	0.9	1.38	1.9	3.7	**0.001**	0.003	**1.5**
C4	0.69	2.9	1.2	0.24	0.26	0.5	0.86	2.0	2.2	0.04	0.05	0.9
T8	0.52	0.6	0.9	0.39	0.39	0.3	0.57	1.7	1.7	0.10	0.11	0.6

The significant differences remaining significant after FDR correction (n = 10 comparisons; *P* < 0.05) are highlighted in bold.

Following this first analysis, we then contrasted the ERPs elicited by statistically legal and illegal words separately in each condition. We compared the mean amplitudes for illegal and legal words in each time-windows (300–400 and 800–900 ms), corresponding to the activity elicited by each of these two syllabic order changes. In order to evaluate these effects, we performed a repeated-measure ANOVA including the factors item type (legal vs. illegal words) and electrode (16 levels) for each time window and condition separately. No significant differences were found in the flat contour condition for illegal words in neither of the two time-windows (1^st^ time-window: item type: *F*(1,25) = 0.35*; P* = 0.56; ƞ_p_^2^ = 0.01; item type by electrode: *F*(1,25) = 1.08*; P* = 0.36; ƞ_p_^2^ = 0.04; 2^nd^ time-window: item type: *F*(1,25) = 0.16*; P* = 0.69; ƞ_p_^2^ = 0.01; item type by electrode: *F*(1,25) = 0.93*; P* = 0.52; ƞ_p_^2^ = 0.04). In contrast, for the melodically enriched condition, a clear positivity was found for illegal words occurring at the first and third syllable order violations (see Fig. 3). Both positive ERP components were significantly larger for illegal than for legal words (1^st^ time-window: item type: *F*(1,25) = 4.81*; P* = 0.03; ƞ_p_^2^ = 0.16; item type by electrode: *F*(1,25) = 1.20*; P* = 0.31; ƞ_p_^2^ = 0.05; 2^nd^ time-window: item type: *F*(1,25) = 13.16*; P* = 0.001; ƞ_p_^2^ = 0.34; item type by electrode: *F*(15,375) = 2.41*; P* = 0.07; ƞ_p_^2^ = 0.08). Because of the almost significant item type by electrode interaction for the second time-window, we performed post-hoc LSD tests and found that illegal words elicited larger positive ERP component than legal words over the 8 fronto-central electrodes (Fp1, Fp2, F3, Fz, F4, C3, Cz, C4; all P’s < 0.01).

We also directly compared the difference waveforms (illegal minus legal words) between the two conditions separately for the first and second time-windows. These difference waveforms were submitted to a repeated-measures ANOVA with the factor condition (flat contour *vs*. melodically enriched) and electrode (16 levels). Compared to the flat contour condition, we found significantly enhanced brain responses in the melodically enriched for the first (condition: *F*(1,25) = 4.33*; P* = 0.04; ƞ_p_^2^ = 0.15; condition by electrode, *F*(15,375) = 1.21*; P* = 0.31; ƞ_p_^2^ = 0.05) and the second time-window (condition: *F*(1,25) = 9.34*; P* = 0.005; ƞ_p_^2^ = 0.27; condition by electrode: *F*(15,375) = 2.36*; P* = 0.06; ƞ_p_^2^ = 0.09).

### Cognitive development and language outcomes at 18 months

Cognitive and language outcome measures could only be obtained from thirteen infants from the original sample (see Method section). The mean productive vocabulary size from the MCDI was 48.8 words (Mdn = 38; percentile 35; SD = 45.07). Cognitive and language attainment assessed with the BSID-III yielded mean standardized scores of 113 (Mdn = 115; SD = 10.07) and 102 (Mdn = 97; SD = 11.82) for the cognitive and the language subscales respectively. Importantly, both measures were within the normal range (100 +/− 15).

### Relationship between neonatal brain responses and expressive vocabulary at 18 months

We first explored the correlations between the relevant EEG measures gathered at 2 days-old in the segmentation task (learning and test phases) and the measures of expressive vocabulary at 18 months (raw MCDI scores and the language scores of the BSID-III). Specifically, considering previous ERP results described above, we selected four EEG measures as possible predictors: (i) the learning brain dynamics reflected by the difference waveforms (block 2 minus block 1) of the learning phases for both the flat and melodically enriched conditions (L_flat and L_melody) in the 200–500 ms time-window, and (ii) for the melodically enriched condition, the difference waveforms obtained by subtracting illegal minus legal word-forms conditions for the two syllabic order changes (300–400 and 800–900 ms time-windows: TW1_melody and TW2_melody).

**Figure 4:**
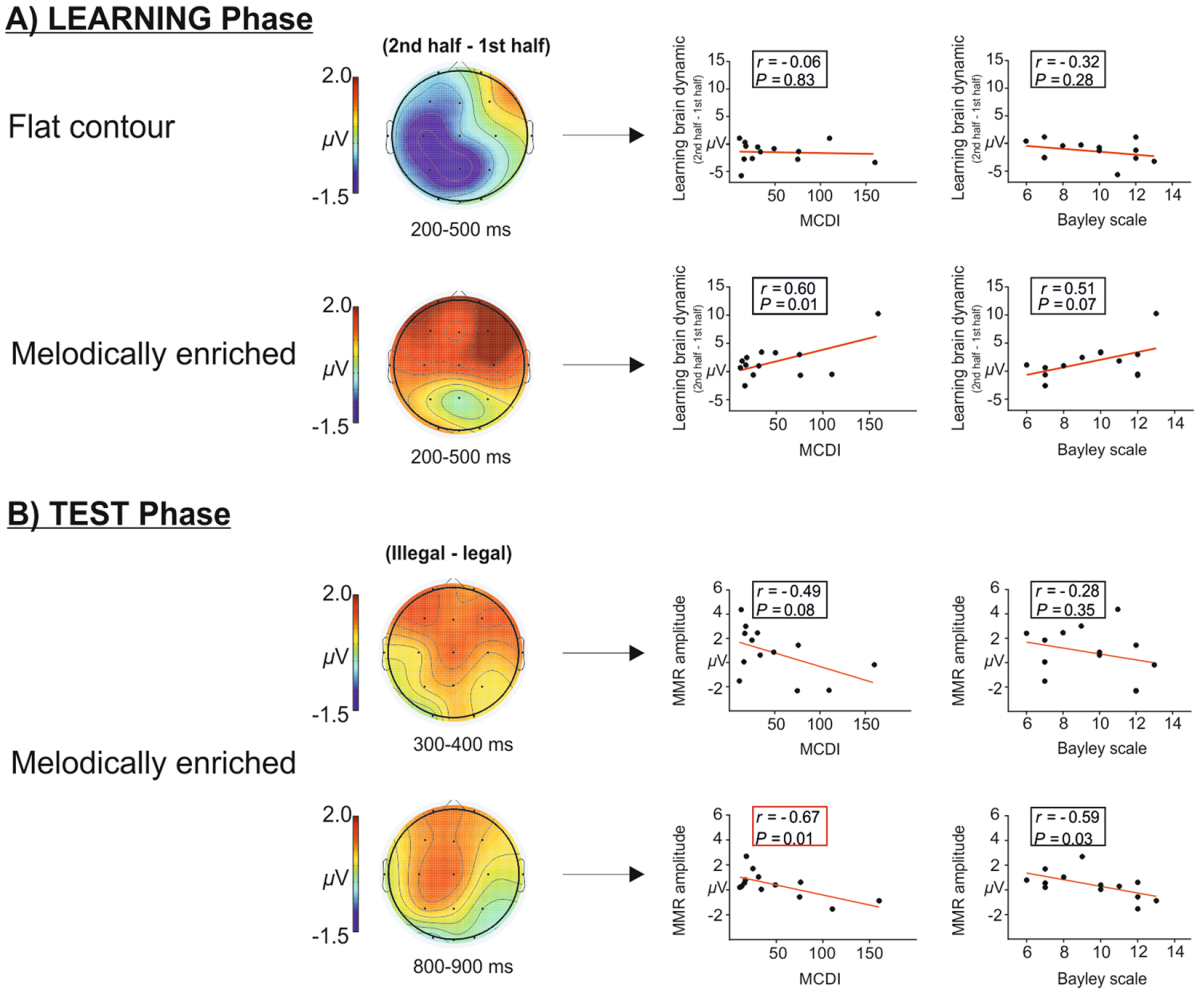
Link between neonatal brain responses in the learning and test phases and expressive vocabulary measures obtained from the MCDI and the Bayley-III language subscale. (**A**) the scatter plots show the correlations between both the raw MCDI scores and the language subscale score of the Bayley-III and the learning brain dynamics averaged across all channels and for each condition. A marginally significant correlation is observed between MCDI scores and the learning brain dynamics in the melodically enriched condition after FDR correction (n = 4 comparisons, *P* < 0.05). (**B**) the scatter plots show the correlations between the raw MCDI scores and the Bayley-III language subscale score and the mean amplitude of the difference waveform (illegal minus legal words) for the two time-windows averaged across all EEG channels. The correlation between the MCDI scores and the second MMR in the melodically enriched condition survives the FDR correction (n = 4 comparisons, *P* < 0.05) and is highlighted in red.

For the ERP variables of the learning phases, the correlation between the MCDI score with the learning effect in the melodically enriched condition (L_melody) was almost significant after FDR correction (r(11) = 0.60, *P*_FDR_ = 0.06). This was not the case in the flat contour condition (see Fig. 4). These results were confirmed by a marginally significant correlation with the language subscale score of the BSID-III (r(11) = 0.51, *P*_FDR_ = 0.14; see Fig. 4) in the melodically enriched condition and not in the flat contour one.

For the two ERP variables of the test phase, we found a significant correlation after FDR correction between the second syllabic order change (TW2_melody) and the MCDI scores (MCDI: r(11) = −0.60, *P*_FDR_ = 0.04, see Fig. 4). This result was confirmed by a marginally significant correlation with the language subscale score of the BSID-III (r(11) = −0.59, *P*_FDR_ = 0.06).

As a second step, we performed a backward multiple regression analysis introducing the four ERP measures as possible predictors of the MCDI scores^41^. In the first model with the four ERP measures included, the predictors accounted for 64% of the variance in expressive vocabulary (*R* ^2^ = 0.64; *F*(4,8) = 3.59, *P* = 0.05). The final model retained only two predictors from the melodically enriched condition: the learning effect and the second violation effect. This model explained 63% of variance and was highly significant (*R* ^2^ = 0.63; *F*(2,10) = 8.38, *P* = 0.007; see Table 2 and Fig. 4).

**Table 2:** Coefficients for the multiple regression with the four neonatal EEG features as predictors (N = 13).

Model	Unstandardized coefficients		Standardized coefficients		
	B	SE	βk	*t*	*P*
*Model 1*					
(Constant)	49.098	13.378		3.595	0.007
L_flat	0.212	5.844	0.009	0.036	0.97
L_melody	6.684	3.291	0.465	2.031	0.07
TW1_melody	−3.822	7.503	−0.174	−0.509	0.62
TW2_melody	−17.552	13.821	−0.421	−1.270	0.24
*Final Model*					
(Constant)	47.575	10.800		4.405	0.001
L_melody	6.263	2.896	0.436	2.163	0.05
TW2_melody	−22.929	8.403	−0.550	−2.729	0.02

The abbreviations L_flat and L_melody correspond to the learning brain dynamics (block 2 minus block 1) in the flat contour and melodically enriched condition respectively. Viol_melody1 and 2 correspond to the difference waveform mean amplitude in the first and second time-windows for the melodically enriched condition (see Methods).

### Cross-validation and generalization

Finally, we examined the generalizability of the results obtained in the multiple regression analysis using a cross-validation strategy based on a machine learning evaluation framework (see Methods). Specifically, we retained the two significant EEG features – the learning brain dynamic (block 2 minus block 1) and the second violation effect (illegal minus legal words) of the melodically enriched condition – in order to further quantify the out-of-sample generalization power of such multiple regression as a predictive model of the MCDI score. Our leave-two-subjects-out cross-validation yielded 156 predicted MCDI scores, which we compared to the true scores. The two complementary evaluation criteria that we computed (*R* ^2^ of the linear regression, 0.56, and mean absolute error, 34.06) were found to be significant when compared to their empirical null distribution estimated using 5000 permutations (*P* = 0.046 and *P* = 0.042 respectively). This analysis directly demonstrates the potential of these two EEG features as biomarkers of future acquired vocabulary.

## Discussion

**Figure 5:**
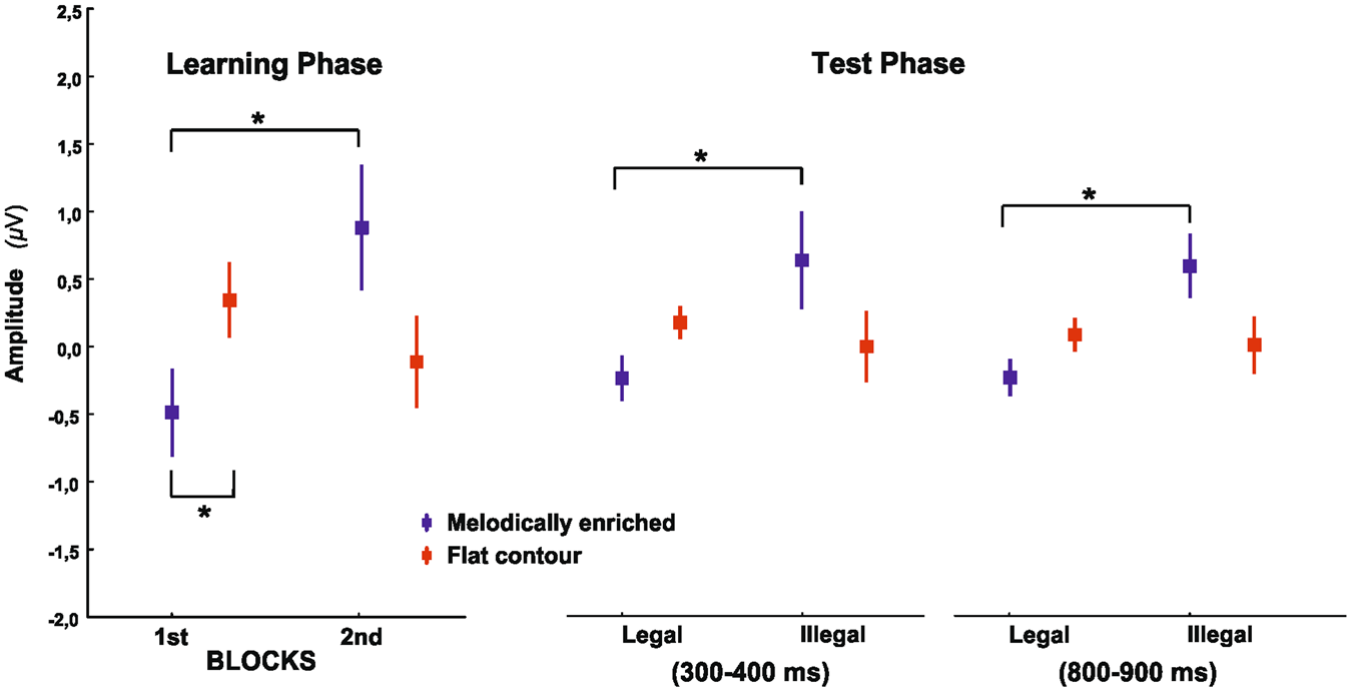
Results of the learning phases (Left): Learning modulations of the ERP mean amplitude in the 200–500 ms latency band for the flat contour (red bars) and melodically enriched conditions (blue bars). Bars denote average amplitudes across all channels (with SEMs) in the two learning blocks. An opposite pattern of ERP modulation is observed in the melodically enriched as compared to the flat contour condition. Results of the test phases (Right): Bars denote average brain response in the 300–400 and 800–900 ms latency band (with SEMs) for legal and illegal word. Significant differences were found in the melodically enriched condition only (blue bars).

The present study reveals in newborns the electrophysiological brain dynamics during statistical learning of flat contour and melodically enriched speech streams. Our results indicate that during the learning phase, melodically enriched and flat contour streams elicited different ERP modulations through exposure. While flat contour streams elicited a positivity during the first block followed by a negativity during the second block, the opposite pattern was observed for the melodically enriched condition. Importantly, brain responses obtained during the implicit test phase revealed successful detection of statistical violations in the melodically enriched but not in the flat contour condition (see Fig. 5). Moreover, using a longitudinal design, we provide empirical and computational evidence for a link between the enhanced brain responses to melodically enriched streams (prosodic modulations) and later expressive vocabulary measures at 18 months.

Previous studies on the building of early word-form memory traces in 6- to 20-month-old infants have consistently revealed a frontal negativity in the 200–500 ms time-window whose amplitude increased with word familiarity. This frontal component has been taken to reflect the emergence of word-form memory traces in infants^40,42^. Additionally, adult studies on speech segmentation have delineated the brain dynamics occurring during the learning phases^30–33^. Results of these studies have consistently revealed modulations of both a P2 and a fronto-central N400-like component through exposure to the streams. Taken together, our results extend previous research^25^ by showing that newborns can already extract word-forms from continuous speech based on statistical cues, as they already exhibit the characteristic brain response indexing the emergence of proto-lexical memory traces^43^. Interestingly, the learning brain dynamics observed in adult participants are tightly linked to the behavioural recognition of the word-forms embedded in the speech streams: results have shown that good learners exhibit a fast increase of the fronto-cental N400-like amplitude after two minutes, while poorer learners showed a slower increase of this negativity through exposure^30,32^. In neonates, different patterns of positive and negative ERP modulation have been observed during exposure to the statistical auditory streams^24,25,29^. Here, we observed a fronto-central negative component during the first half of the learning phase followed by a broad positivity during the second half in the melodically enriched condition. Surprisingly, an opposite pattern of ERP modulation was found in the flat contour condition with a positivity followed by a negativity through stream exposure. Nonetheless, the longitudinal design used here provides additional evidence for the importance of the learning brain dynamics during speech segmentation. Indeed, the only significant relationships between neonatal brain responses and expressive vocabulary at 18 months were found for the melodically enriched condition only (see Table 2). The predictive power of these potential biomarkers related to language abilities was further confirmed by the machine learning approach we used here. Therefore, considering previous results on speech segmentation in adults, as well as results on word familiarity in infants showing similar negativities at similar latencies^25,40,42^, our results suggest that melodic information does indeed facilitate speech segmentation in neonates by enabling a more efficient extraction of word-forms.

However, none of the previous studies we have discussed have assessed the ability to detect on-line statistical violations after learning had taken place. This is crucial because during the learning phase of such statistical learning paradigms, only sufficiently reinforced items may survive interference and be stored in long-term memory^44^. Considering this constraint and the fact that behavioural measures are difficult to collect at a very early age (in 2-day-old infants), we sought neural evidence of online detection of structural violations that may indicate the building of sufficiently robust word-form memory traces. During the implicit test phase, illegal word-forms induced enhanced brain responses for the violations occurring at the first and last syllable positions in the melodically enriched condition only. No significant difference was encountered in the flat contour condition. While a fronto-central negative MMR has been observed in response to similar statistical violations in adults^30^, a positive MMR has been systematically reported in newborns for different types of auditory deviants including simple and complex pitch regularities^45,46^. This component is also observed for speech sound changes^1,47^ and its amplitude predicts later language development^48,49^. Taken together, the results of the test phase confirmed that melodically enriched streams facilitated the building of robust word-form memory traces and triggered strong enough expectations to successfully detect structural violations of the extracted word-forms in the test phase. In addition, these results further confirmed the importance of brain dynamics observed during the learning phase, as neonates were sensitive to changes in the statistical structure only for the melodically enriched streams. In sum, while brain signatures of successful speech segmentation were observed during the learning phase in both conditions (albeit with different dynamics), successful retention (i.e. the building of memory traces) of the extracted word-forms only occurred in the presence of melodic cues (as revealed in the test phase). Therefore, the intriguing question of why musical cues can boost speech segmentation needs to be addressed.

Compared to adult-direct speech, infant-directed speech is notably characterized by a slower rate of speech, a higher fundamental frequency and by the repetition of intonation structures^50,51^. In the present study, the repetition of the 3-tone pitch patterns in the melodically enriched streams contained systematic pitch-contour and pitch interval changes at word boundaries that may have aided the grouping of syllables^52^. Additionally, the syllables and pitches were correlated in the melodically enriched condition which may explain the enhanced brain responses for illegal words in the test phase. This would be in line with the observation of faster learning when a second source of information can provide redundant cues to identify the structure of the input^53^. Future studies are needed to determine whether newborns used both cues or deviants in pitch pattern alone. An alternative explanation might be related to the fact that melodically enriched streams, which contain pitch contour variations, have been found to enhance overall attention and emotion in infants^50,51,54–56^. For instance, compared to adult-directed speech stimuli, young infants prefer to listen to IDS probably due to its higher capacity to maintain infants’ attention or convey emotional information^54,56^. Considering the similarities between infant-directed speech and music in terms of pitch and tempo modulations, as well as the impressive capacity of music to convey emotions^57,58^, songs could act in neonates as an innate catalyst of exogenous attention and emotion. In this context, it is well known that memory encoding is facilitated with high levels of attention and emotional processing^59,60^. This hypothesis is also consistent with behavioural data showing unsuccessful learning in infants when exposed to an impoverished emotional infant-directed speech^61^. Therefore, the presence of melodic cues in our experiment could have induced stronger attentional or reward-related processes promoting word-form extraction through the activation of reinforcement learning mechanisms. Indeed, the important role of emotion and reward in word learning and auditory learning in general has been recently proposed^62,63^. Moreover, it has also been observed that adults and infants could associate novel word-forms to objects more easily when presented with material containing the rich prosodic characteristics of IDS rather than with ADS^18–20^. Interestingly, it has been recently demonstrated that 12- and 24-months old infants’ brain responses of word recognition is mediated by increased attention allocation through predictive coding processes^64^. Besides, music processing *per se* as well as the rewarding experience of music may also rely on predictive coding mechanisms^57^. Therefore, increased attentional and reward-related processing during exposure to melodically enriched streams might explain, at least in part, the facilitating effects observed in our data.

Nonetheless, the present study shows several limitations that must be mentioned. Further studies are needed to determine whether increasing the duration of the exposure phase in the flat contour condition may induce a similar dynamic evolution of the amplitude of the fronto-central negativity, as it is the case in the melodically enriched condition. Besides, it is important to note that the melodically enriched streams used here contained syllables that were “sung” in a musical structure with 11 notes out of 12 belonging to the C major scale, which may have induced the perception of a tonal centre^65^ and could also have consequences on the formant information (e.g. F1, F2, and F3) which could be additional information to word segmentation. Indeed, newborns are sensitive to western musical chords and exhibit a large positive MMR to dissonant chords in a sequence of chords from the C major scale^46^. Further studies using different musical scales thus needed to determine the role of the tonality in triggering word-form extraction in early infancy as well as the role of formant information. Another limitation of the present study concerns the electrophysiology method and the use of the average mastoids as reference. In the present study, we performed an off-line referencing of our EEG data to the average mastoids in order to make our results easily comparable with previous reports on speech segmentation in neonates^25,29,64^ and adults^26,27,30–33,65,66^. As recent studies using simulated and experimental data have suggested that the use of infinite reference based on the reference electrode standardization technique can avoid scalp topography distortions and localization errors^67,68^, future studies in neonates should compare the different methods of re-referencing. Another limitation to acknowledge concerns the comparisons of the two conditions, flat contour and melodically enriched. As compared to the flat contour streams, the melodically enriched streams contained pitch modulations that may have contaminated the ERP results, specifically when it comes to compare the difference waves between conditions. Moreover, the babies included in the present study did not present any hearing-related dysfunctions as all of them had normal results in the screening procedure performed during the first weeks of life using Automated Auditory Brainstem Responses. Data obtained about familiar history of dyslexia (only 86% of the families provided information on this issue), showed that the father of one of the infants had been diagnosed as dyslexic which is a factor known to influence early brain responses to speech sounds^48,49^. It will be important for future studies to take this parameter into account. Besides this, although an important percentage of the infants (62%) enrolled in the present study were mostly raised in monolingual families, some of them had been prenatally exposed to both Spanish and Catalan from their bilingual mothers. There is evidence showing differences in speech perception abilities between monolingual and bilingual infants early in development^69–71^. More crucial for the present study, sensitivity to differential language properties has been found to start even before birth in newborns whose mothers had regularly spoken two rhythmically different languages during pregnancy^72^. Our bilingual infant participants had been prenatally exposed to two rhythmically close languages and evidence for robust intraclass language discrimination at birth is still lacking. However, an emerging early sensitivity to specific rhythm properties of the languages or attention to any differential features that might be captured before birth cannot be totally discarded in bilingual newborns, in contrast to those exposed prenatally to a single language. These differences may certainly start the last trimester of gestation when the cortical plate is setting up^5,6^. Therefore, it will be important to take these language exposure parameters into account in future studies.

Some practical considerations have also to be acknowledged. Firstly, we used a fixed order of presentation with the flat contour condition always presented first. This was done in order to reduce any possible facilitation effect carried by the melodically enriched condition. Moreover, we used different languages built with different CV syllables with no repetition of the same syllable across conditions to control for possible effect of interference between the two conditions. Even if we cannot clearly rule-out any carry-over effect from the first to the second condition, there is evidence, at least in adults, that learning a first artificial language decreases the capacity to learn the second one^73^. Future studies are necessary to further disentangle the possible effect of presentation order. Finally, in spite of a longitudinal design, vocabulary measures at 18 months could only be gathered for 13 of the participants in the original sample, a factor that constrains the statistical power of our analyses. Nonetheless, results of the machine learning evaluation procedure used here clearly demonstrated the importance of neonatal brain sensitivity to melodically enriched as compared to flat contour streams in later vocabulary acquisition. Further longitudinal studies with larger samples minimizing the effects of the observed attrition rate in these studies are needed to provide further support for the current data and to clearly characterize the link between neonatal brain sensitivity to prosodic modulations and later language abilities.

In summary, we found brain signatures of successful word-form segmentation for both melodically enriched and flat contour streams in 2-day-old neonates. The brain dynamics were different in the two conditions but crucially, neural responses of successful detection of structural violations was found in the melodically enriched condition only, indicating that sung streams induced a more efficient learning. Importantly, the longitudinal design used here offers a unique opportunity based on empirical and computational evidence to establish a link between newborns’ brain sensitivity to melodically enriched speech streams and expressive vocabulary at 18 months. Previous results in adults and children have shown that musical training enhances speech segmentation^65,66^. Moreover, musical intervention at 9 months of age enhances brain sensitivity to the temporal structure of music and speech^74^. Taken together, our results converge in showing the importance of musically enriched input in the early steps of language acquisition. These findings may thus have clear implications for the important role of music-based programs on language learning.

## Methods

### Participants

Twenty-eight healthy full-term neonates (mean age at testing = 2.8 days, SD = 0.9, range: 1–4 days; 14 boys; mean birth weight = 3375 g; SD = 506.4, range: 2640–4420 g; the mean Apgar score obtained at 5 minutes of life was >8 and the mean Apgar score obtained at 10 minutes of life was >9; mean gestational age = 40 weeks; SD = 0.8, range: 37–41 weeks) were enrolled in this experiment. Note that the Apgar score is an objective tool that measures different signs of physiologic adaptation after birth. An Apgar score of 7 or more indicates that the baby is in good condition. Newborns were recruited and tested at Hospital Sant Joan de Déu, Barcelona, Spain. Only infants born from parents whose native language was either Spanish or Catalan participated in the study. Information about their native language, the language predominantly used at home and an estimate of the daily use of each of the ambient languages (i.e. Spanish and Catalan) by the mother in the last three months of pregnancy was obtained through an interview at the hospital room. Although all parents could speak and understand both Catalan and Spanish, the interview revealed that 62% of the families had a predominant language use (above 75% of regular use of only Spanish or Catalan), the remaining families showing a more clear bilingual status in the daily use of the two languages present in the community. Parents were informed and signed a consent form at the beginning of the experimental session. This study was carried out in accordance with the guidelines of the Declaration of Helsinki (BMJ 1991; 302: 1194) and approved by the Ethical Committee of Hospital Sant Joan de Déu (University of Barcelona; CEIC-PIC-69-13). All neonates passed a hearing screening test (ABBR, Automated Auditory Brainstem Response) and an examination by a neonatologist at the delivery ward.

### Stimuli

#### Learning streams

Two languages (L1 and L2) were built using a set of 24 synthetic syllables that were combined to give rise to two sets of four tri-syllabic pseudo-words (L1: KUPOTE, DOGUKI, TAPIKO, DAGOPE; L2: TUGODE, PAGODI, DUGAKE, KATIPU). In both conditions, the syllable duration was set to 350 ms thus leading to 1050 ms tri-syllabic pseudo-words. In the flat contour condition, all the syllables had the same pitch of 216 Hz. In the melodically enriched condition and for each language, each of the 12 syllables was associated with a distinct tone. Therefore each word had a unique melodic contour (KUPOTE: D_4_C_4_G_3_, DOGUKI: D^b^_4_A_3_E_3_, TAPIKO: B_3_E_4_F_4_, DAGOPE: C_3_D_3_F_3_). No pitch-contour changes occurred within the pseudo-words (2 pseudo-words contained a rising pitch-contour while 2 contained a falling pitch-contour). The mean pitch interval within pseudo-words was significantly different from the mean interval between the pseudo-words (*P* = 0.02). Moreover, pitch-contour changes could be used to segment the stream, as this cue was consistent and no other acoustic cues were inserted at word boundaries. The four learning streams were built by a random concatenation of the four words (without repetition of the same item twice in a row). All the stimuli were synthesized using Mbrola (http://tcts.fpms.ac.be/synthesis/mbrola.html) with the Spanish es2 database and thus were only built with syllables that were part of the Spanish-Catalan phonetic repertoire. The transitional probability between the syllables forming a word was 1.0, while the transitional probability between syllables spanning word boundaries was 0.33. Each word was repeated 50 times in the stream leading to 3.5 minutes continuous speech streams. Importantly, no repetitions of syllables were made across the two language streams (L1 and L2).

#### Tests streams

The test streams consisted of 66 repetitions of each pseudo-word from the corresponding language in which test items were pseudo-randomly inserted as done previous studies^30,31^. The test items were built by reversing the order of the syllables composing each of the words. The test items were TEPOKU, KIGUDO, KOPITA and PEGODA for L1 and DEGOTU, DIGOPA, KEGADU, PUTIKA for L2. The test items were inserted in the continuous stream but could not follow the word from which it was derived. The overall probability of the test items in the test streams was 25% (22 repetitions of each novel pseudo-words).

### Experimental procedure and data acquisition

The entire recording session took place directly in the room of the maternity unit. During the experimental session, infants were lying asleep on their cribs while being presented with the auditory streams (learning phase and implicit test) of both conditions in a within-subject design. All infants were presented with the flat contour condition followed by the melodically enriched condition. Half of the infants were exposed to one language in the flat contour and to the other in the melodically enriched condition and this was counter-balanced across participants. At least one of the 2 parents was present during the session. Stimuli were presented at a 70-dB volume from one loudspeaker positioned in front of the infants at a distance of about 1 m from the infant’s crib. EEG was recorded from 16 scalp electrodes (Biosemi ActiveTwo system, Amsterdam University) located at standard positions (International 10/20 system sites: Fp1, Fp2, F3, F4, T7, C3, C4, T8, P3, P4, O1, O2, Fz, Cz, Pz and Oz). The data of three newborns had to be discarded due to excessive movements inducing major EEG artifacts. The EEG was amplified by Biosemi amplifiers with a band-pass of 0–102.4 Hz and was digitized at 250 Hz.

### Vocabulary and cognitive measures

At 18 months, parents of the participants were contacted again and asked to come to the Lab for a language and cognitive development assessment. Parents were requested to fill out the expressive vocabulary checklist form the Spanish version of the MacArthur-Bates Communicative Development Inventory^75^ (MCDI). This questionnaire provided us with a parental estimate of expressive vocabulary size. Infants were also tested with the Bayley Scales of Infant Development^76^ (BSID-III). Only the Cognitive and the Language subscales were used. A general measure of cognitive development was obtained to ensure that all participants in the sample fell within the normal range. The Language subscale score was obtained as a more standard measure of toddlers’ attainment in both receptive and expressive language development. We expected the latter measure to positively correlate with the expressive vocabulary reports from the MCDI, thus limiting the presence of parental biases in the way they reported toddlers’ spontaneous word productions. Both the BSID-III cognitive and language subscales and the MCDI questionnaire were administered in a room of the APAL Infant Lab at the Hospital Sant Joan de Déu. The data of thirteen 18 month-olds could be satisfactorily gathered.

### EEG data analyses

All the EEG data were processed and analyzed using custom scripts programmed in Matlab (version R2008b) and with functions of the EEGLAB toolbox. For the learning phases, EEG data were re-referenced offline to the left and right mastoidal electrodes and were filtered offline using a 0.5–20 Hz bandpass filter (FIR, Hamming window, max stopband attenuation = −53 dB, Max passband deviation = 0.0063; as done in previous reports studying the EEG correlates of the learning phases^24,25,30,32^). Continuous EEG data were then split into epochs from −50 ms to 1050 ms from word onset, baseline-corrected and epochs containing external artifacts exceeding ±120 μV were automatically removed from analyses. ERP data were analyzed by computing the mean amplitudes between 200 and 500 ms post word-onset independently of the sleep stage of the newborns. This time window was selected based on visual inspection and previous findings with statistical learning paradigms in infants and adults^24,25,32,33^. We used repeated-measures analysis of variance (ANOVA) with condition (flat contour vs. melodically enriched), blocks (first vs. second, based on 2 consecutive non-overlapping time windows of 1′45″, 100 trials each) and electrodes (16 levels) as within-subject factors.

For each ANOVA, Greenhouse-Geisser sphericity corrections were applied when appropriate. Partial-eta-squared (ηp2) was computed for the main effects and interactions with other factors. Post-hoc tests were conducted using the Fisher LSD method as done in^25^. Cohen’s d effect sizes were also computed and reported when appropriate.

For the test phases, EEG data were re-referenced offline to the left and right mastoidal electrodes and were offline filtered from 1 to 20 Hz (as done in previous auditory MMR studies in newborns and children^35,77–79^). Continuous EEG data were then split into epochs from −50 ms to 1050 ms from word onset, baseline-corrected and epochs containing external artifacts exceeding ±120 μV were automatically removed from analyses. Firstly, in order to detect the presence of a MMR, two-tailed Student’s *t*-tests were used to compare the mean amplitude of the difference waveform against zero in each condition. Based on the results of this first exploratory analysis and on previous studies using similar types of deviants^30^, ERP data were analyzed separately for each condition by comparing the mean amplitudes for illegal and legal words in two time-windows (300–400 and 800–900 ms) covering the activity elicited by the two statistical violations (see^30^ for similar analyses in adults). Finally, in a third step analysis that aimed to directly compare the two conditions in the two time-windows of interest, the difference waveforms of the two conditions (illegal minus legal words) were submitted to a repeated-measures ANOVA with the factor condition (flat contour *vs*. melodically enriched) and electrode (16 levels). As done for the learning phases, Greenhouse-Geisser sphericity corrections were applied when appropriate. Partial-eta-squared (η_p_^2^) was computed for the main effects and interactions with other factors. Post-hoc tests were conducted using the Fisher LSD method as done in^25^. Cohen’s d effect sizes were also computed and reported when appropriate.

### Relationship between expressive vocabulary at 18 months and neonatal brain responses

As a first exploratory step, we assessed the linear relationship between the EEG measures gathered during the learning phases and the measures of expressive vocabulary at 18 months (raw MCDI scores and the language scores of the BSID-III). Considering the ERP results of the learning phases, we used the learning brain dynamics reflected by the mean amplitude averaged across all EEG channels for the 200–500 ms time-window of difference waveforms (block 2 minus block 1) in the learning phases for both the flat and melodically enriched conditions (L_flat and L_melody). The Pearson correlations between the difference waveforms (block 2 minus block 1) of the learning phases relative to the raw MCDI scores and the language scores of the BSID-III were evaluated. The correlations were considered significant for *P*-values lower than 0.0125 (after FDR correction with n = 4 comparisons).

To investigate the relationship between the EEG measures gathered during the test phases and the measures of expressive vocabulary at 18 months we only considered the two MMR occurring at each syllabic violation (difference illegal minus legal words in the 300–400 and 800–900 ms time-windows averaged across all EEG channels) in the melodically enriched condition. This choice was driven by the absence of significant MMR in this condition only. The Pearson correlations between the difference waveforms obtained by subtracting illegal minus legal word-forms conditions for the two syllabic order changes (300–400 and 800–900 ms time-windows: TW1_melody and TW2_melody) relative to the raw MCDI scores and the language scores of the BSID-III were evaluated. The correlations were considered significant for *P*-values lower than 0.0125 (after FDR correction with n = 4 comparisons).

As a second step, we performed a backward multiple linear regression to explore the possible predictive value and further confirm the contribution of each of the different EEG features gathered at two days after birth on the language outcomes obtained at 18 months. Specifically, we focused on expressive vocabulary measures, a variable that has been reported to significantly correlate with the speech segmentation skill assessed prelexically^38–40^. We used four neonatal EEG features to predict the MCDI raw scores. For the learning phases of both conditions, we considered the mean amplitude averaged across all EEG channels for the 200–500 ms time-window of the difference waveform (block 2 minus block 1). For the test phase, because significant MMR were found in the melodically enriched condition only, we considered the two MMR occurring at each syllabic violation (difference illegal minus legal words in the 300–400 and 800–900 ms time-windows averaged across all EEG channels).

Following this analysis, we explicitly quantified the predictive power of the significant EEG features using a machine learning evaluation framework^80^. For this, we removed two subjects from the dataset, fitted the same model on the eleven other subjects and used it to obtain a predicted MCDI value from the EEG features of the two left-out subjects. This procedure was repeated throughout a cross-validation procedure^81^ (leave-two-subjects-out) where all 78 combinations of two-out-of-thirteen newborns were used to obtain out-of-sample predicted MCDI scores – thus allowing to obtain a more robust result than with a leave-one-subject-out scheme. We then gathered all the predicted scores and evaluated their similarity to the true scores by both performing a linear regression and computing the mean absolute error. The statistical significance of this evaluation was assessed in a non-parametric way using a permutation test^82^, which allows estimating the empirical distribution of the computed scores under the null hypothesis of no effect. This statistical procedure was necessary to account for the non-independence of the predicted scores, induced by the cross validation.

## Electronic supplementary material

Supplementary figures.

## References

[1] Partanen E (2013). Learning-induced neural plasticity of speech processing before birth. Proc. Natl. Acad. Sci. USA.

[2] Benavides-Varela S, Hochmann JR, Macagno F, Nespor M, Mehler J (2012). Newborn’s brain activity signals the origin of word memories. Proc. Natl. Acad. Sci. USA.

[3] Lecanuet JP, Graniere-Deferre C, Jacquet AY, DeCasper AJ (2000). Fetal discrimination of low-pitched musical notes. Dev. Psychobiol..

[4] Partanen E, Kujala T, Tervaniemi M, Huotilainen M (2013). Prenatal music exposure induces long-term neural effects. PloS ONE.

[5] Rakic P (1988). Specification of cerebral cortical areas. Science.

[6] Rakic P (1995). A small step for the cell, a giant leap for mankind: a hypothesis of neocortical expansion during evolution. Trends Neurosci..

[7] Dehaene-Lambertz G, Dehaene S, Hertz-Pannier L (2002). Functional neuroimaging of speech perception in infants. Science.

[8] Dehaene-Lambertz G (2006). Functional organization of perisylvian activation during presentation of sentences in preverbal infants. Proc. Natl. Acad. Sci. USA.

[9] Perani D (2011). Neural language networks at birth. Proc. Natl. Acad. Sci. USA.

[10] Perani D (2010). Functional specializations for music processing in the human newborn brain. Proc. Natl. Acad. Sci. USA.

[11] Telkemeyer S (2009). Sensitivity of newborn auditory cortex to the temporal structure of sounds. Journal of Neuroscience.

[12] Zatorre RJ, Belin P (2001). Spectral and temporal processing in human auditory cortex. Cerebral Cortex.

[13] Giraud AL (2007). Endogenous cortical rhythms determine cerebral specialization for speech perception and production. Neuron.

[14] Winkler I, Háden GP, Ladinig O, Sziller I, Honing H (2009). Newborn infants detect the beat in music. Proc. Natl. Acad. Sci. USA.

[15] Nazzi T, Floccia C, Bertoncini J (1998). Discrimination of pitch contours by neonates. Infant Behav. and Dev..

[16] Granier-Deferre C, Ribeiro A, Jacquet AY, Bassereau S (2011). Near-term fetuses process temporal features of speech. Dev. Sci..

[17] Mampe B, Friederici AD, Christophe A, Wermke K (2009). Newborns’ cry melody is shaped by their native language. Curr. Biol..

[18] Shukla M, White KS, Aslin RN (2011). Prosody guides the rapid mapping of auditory word forms onto visual objects in 6-mo-old infants. Proc. Natl. Acad. Sci. USA.

[19] Trainor LJ, Desjardins RN (2002). Pitch characteristics of infant-directed speech affect infants’ ability to discriminate vowels. Psychonomic Bulletin and Review.

[20] Thiessen ED, Hill E, Saffran JR (2005). Infant-directed speech facilitates word segmentation. Infancy.

[21] Saffran JR, Aslin RN, Newport EL (1996). Statistical learning by 8-month-old infants. Science.

[22] Thiessen ED, Saffran JR (2003). When cues collide: Use of stress and statistical cues to word boundaries by 7- to 9-month-old infants. Dev. Psychol..

[23] Bulf H, Johnson SP, Valenza E (2011). Visual statistical learning in the newborn infant. Cognition.

[24] Kudo N, Nonaka Y, Mizuno N, Mizuno K, Okanoya K (2011). On-line statistical segmentation of a non-speech auditory stream in neonates as demonstrated by event-related brain potentials. Dev. Sci..

[25] Teinonen T, Fellman V, Näätänen R, Alku P, Huotilainen M (2009). Statistical language learning in neonates revealed by event-related brain potentials. BMC Neuroscience.

[26] Cunillera T, Toro JM, Sebastián-Galles N, Rodríguez-Fornells A (2006). The effects of stress and statistical cues on continuous speech segmentation: an event-related brain potential study. Brain Research.

[27] De Diego Balaguer R, Rodríguez-Fornells A, Bachoud-Lévi AC (2015). Prosodic cues enhance rule learning by changing speech segmentation mechanisms. Front. Psychol..

[28] Johnson EK, Jusczyk PW (2001). Word segmentation by 8-month-olds: When speech cues count more than statistics. J. Mem. and Lang..

[29] Bosseler AN, Teinonen T, Tervaniemi M, Huotilainen M (2016). Infant Directed Speech Enhances Statistical Learning in Newborn Infants: An ERP Study. PloS ONE.

[30] François C, Cunillera T, Garcia E, Laine M, Rodriguez-Fornells A (2017). Neurophysiological evidence for the interplay of speech segmentation and word-referent mapping during novel word learning. Neuropsychologia.

[31] D D Balaguer R, Toro JM, Rodriguez-Fornells A, Bachoud-Lévi AC (2007). Different neurophysiological mechanisms underlying word and rule extraction from speech. PloS ONE.

[32] François C, Jaillet F, Takerkart S, Schön D (2014). Faster sound stream segmentation in musicians than in nonmusicians. PloS ONE.

[33] Cunillera T (2009). Time course and functional neuroanatomy of speech segmentation in adults. NeuroImage.

[34] Alho K, Sainio K, Sajaniemi N, Reinikainen K, Näätänen R (1990). Event-related brain potential of human newborns to pitch change of an acoustic stimulus. Electroencephalography and Clinical Neurophysiology/Evoked Potentials Section.

[35] Háden GP, Németh R, Török M, Winkler I (2015). Predictive processing of pitch trends in newborn infants. Brain research.

[36] Näätänen R, Jacobsen T, Winkler I (2005). Memory‐based or afferent processes in mismatch negativity (MMN): A review of the evidence. Psychophysiology.

[37] Kuhl PK (2004). Early language acquisition: cracking the speech code. Nat. Rev. Neurosci..

[38] Newman R, Ratner NB, Jusczyk AM, Jusczyk PW, Dow KA (2006). Infants’ early ability to segment the conversational speech signal predicts later language development: a retrospective analysis. Dev. Psychol..

[39] Singh L, Steven Reznick J, Xuehua L (2012). Infant word segmentation and childhood vocabulary development: a longitudinal analysis. Dev. Sci..

[40] Junge C, Kooijman V, Hagoort P, Cutler A (2012). Rapid recognition at 10 months as a predictor of language development. Dev. Sci..

[41] Cohen, J., Cohen, P., West, S. G. & Aiken, L. S. Applied multiple regression/correlation analysis for the behavioral sciences. Routledge (2013).

[42] Friedrich M, Friederici AD (2011). Word learning in 6-month-olds: fast encoding-weak retention. J. Cogn. Neuro..

[43] Rodríguez-Fornells A, Cunillera T, Mestres-Missé A, De Diego-Balaguer R (2009). Neurophysiological mechanisms involved in language learning in adults. Philos. Trans. R. Soc. Lond. B. Biol. Sci..

[44] Perruchet P, Vinter A (2002). The self-organizing consciousness. Behav. Brain. Sci..

[45] Stefanics G (2009). Newborn infants process pitch intervals. Clin. Neurophysiol..

[46] Virtala P, Huotilainen M, Partanen E, Fellman V, Tervaniemi M (2013). Newborn infants’ auditory system is sensitive to Western music chord categories. Front. Psychol..

[47] Dehaene-Lambertz G (2000). Cerebral specialization for speech and non-speech stimuli in infants. J. Cogn. Neurosci..

[48] Leppänen PH (2010). Newborn brain event-related potentials revealing atypical processing of sound frequency and the subsequent association with later literacy skills in children with familial dyslexia. Cortex.

[49] Guttorm TK (2005). Brain event-related potentials (ERPs) measured at birth predict later language development in children with and without familial risk for dyslexia. Cortex.

[50] Trehub, S. E., Trainor, L. J. & Unyk, A. M. Music and speech processing in the first year of life. In H.W. Reese (Ed.), Advances in child development and behavior (Vol. 24, pp. 1–35). New York: Academic Press (1993). 10.1016/s0065-2407(08)60298-0 8447246

[51] Fernald, A. Maternal vocalisations to infants as biologically relevant signals: An evolutionary perspective. In Barkow, J. H. Cosmides, L. & Tooby, J. (Eds), The adapted mind: Evolutionary psychology and the generation of culture. Oxford: Oxford University Press (1992).

[52] Schön D (2008). Song as an aid for language acquisition. Cognition.

[53] Christiansen MH, Allen J, Seidenberg MS (1998). Learning to segment speech using multiple cues: a connectionist model. Lang. Cogn. Process..

[54] Fernald A (1985). Four-month-old infants prefer to listen to motherese. Infant Behav. and Dev..

[55] Fernald A (1993). Approval and disapproval: Infant responsiveness to vocal affect in familiar and unfamiliar languages. Child Dev..

[56] Trainor LJ, Austin CM, Desjardins RN (2000). Is infant-directed speech prosody a result of the vocal expression of emotion?. Psychol. Sci..

[57] Blood AJ, Zatorre RJ (2001). Intensely pleasurable responses to music correlate with activity in brain regions implicated in reward and emotion. Proc. Natl. Acad. Sci. USA.

[58] Koelsch S (2010). Towards a neural basis of music-evoked emotions. Trends Cogn. Sci..

[59] Craik FI, Govoni R, Naveh-Benjamin M, Anderson ND (1996). The effects of divided attention on encoding and retrieval processes in human memory. J. Exp. Psychol. Gen..

[60] Adcock RA, Thangavel A, Whitfield-Gabrieli S, Knutson B, Gabrieli JD (2006). Reward-motivated learning: mesolimbic activation precedes memory formation. Neuron.

[61] Kaplan PS, Bachorowski JA, Smoski MJ, Hudenko WJ (2002). Infants of depressed mothers, although competent learners, fail to learn in response to their own mothers’ infant-directed speech. Psychol. Sci..

[62] Ripollés P (2014). The role of reward in word learning and its implications for language acquisition. Curr. Biol..

[63] Kraus N, White-Schwoch T (2015). Unraveling the biology of auditory learning: A Cognitive-Sensorimotor-Reward framework. Trends Cogn. Sci..

[64] Ylinen, S., Bosseler, A., Junttila, K., Huotilainen, M. Predictive coding accelerates word recognition and learning in the early stages of language development. *Dev. Sci*, 10.1111/desc.12472 (2016). 10.1111/desc.12472 27747989

[65] François C, Schön D (2011). Musical expertise boosts implicit learning of both musical and linguistic structures. Cerebral Cortex.

[66] François C, Chobert J, Besson M, Schön D (2013). Music training for the development of speech segmentation. Cerebral Cortex.

[67] Yao D (2017). Is the Surface Potential Integral of a Dipole in a Volume Conductor Always Zero? A Cloud Over the Average Reference of EEG and ERP. Brain Topography.

[68] Liu Q (2015). Estimating a neutral reference for electroencephalographic recordings: the importance of using a high-density montage and a realistic head model. J. neural eng..

[69] Ferjan Ramírez, N., Ramírez, R. R., Clarke, M., Taulu, S., Kuhl, P. K. Speech discrimination in 11‐month‐old bilingual and monolingual infants: a magnetoencephalography study. *Dev. Sci*. **20**(1) (2017). 10.1111/desc.12427 27041494

[70] Bosch L, Sebastián-Gallés N (1997). Native-language recognition abilities in four-month-old infants from monolingual and bilingual environments. Cognition.

[71] Bosch L, Sebastián-Gallés N (2001). Evidence of early language discrimination abilities in infants from bilingual environments. Infancy.

[72] Byers-Heinlein K, Burns TC, Werker JF (2010). The roots of bilingualism in newborns. Psychological science.

[73] Franco A, Cleeremans A, Destrebecqz A (2011). Statistical learning of two artificial languages presented successively: how conscious?. Front. Psychol..

[74] Zhao TC, Kuhl PK (2016). Musical intervention enhances infants’ neural processing of temporal structure in music and speech. Proc. Natl. Acad. Sci. USA.

[75] López-Ornat, S. *et al*. Inventario de Desarrollo Comunicativo MacArthur, adaptación española. Madrid: TEA Ediciones, S.A. (2005).

[76] Bayley, N. Bayley Scales of Infant and Toddler Development. 3rd edn. San Antonio, TX: Harcourt Assessment Inc (2006).

[77] Partanen E, Pakarinen S, Kujala T, Huotilainen M (2013). Infants’ brain responses for speech sound changes in fast multifeature MMN paradigm. Clin. Neurophysiol..

[78] Leipälä JA, Partanen E, Kushnerenko E, Huotilainen M, Fellman V (2011). Perinatal cerebral insults alter auditory event-related potentials. Early human development.

[79] Mahmoudzadeh M, Wallois F, Kongolo G, Goudjil S, Dehaene-Lambertz G (2017). Functional maps at the onset of auditory inputs in very early preterm human neonates. Cerebral Cortex.

[80] Gabrieli JD, Ghosh SS, Whitfield-Gabrieli S (2015). Prediction as a humanitarian and pragmatic contribution from human cognitive neuroscience. Neuron.

[81] Varoquaux G (2017). Assessing and tuning brain decoders: Cross-validation, caveats, and guidelines. NeuroImage.

[82] Anderson MJ, Robinson J (2001). *Permutation Tests* for Linear Models. Australian & New Zealand Journal of Statistics.

