# Supplementary material for: Enhanced Neonatal Brain Responses To Sung Streams Predict Vocabulary Outcomes By Age 18 Months
Source: Sci Rep. 2017 Sep 29;7:12451. doi: 10.1038/s41598-017-12798-2 (PMC5622081; doi:10.1038/s41598-017-12798-2)
Supplement: Supplementary file 1 — Supplementary figures. [file 41598_2017_12798_MOESM1_ESM.doc]

**ENHANCED NEONATAL BRAIN RESPONSES TO SUNG STREAMS PREDICT VOCABULARY OUTCOMES BY AGE 18 MONTHS**

Clément François, Maria Teixidó, Sylvain Takerkart, Thaïs Agut,

Laura Bosch & Antoni Rodriguez-Fornells

**Supplementary figure 1**

**
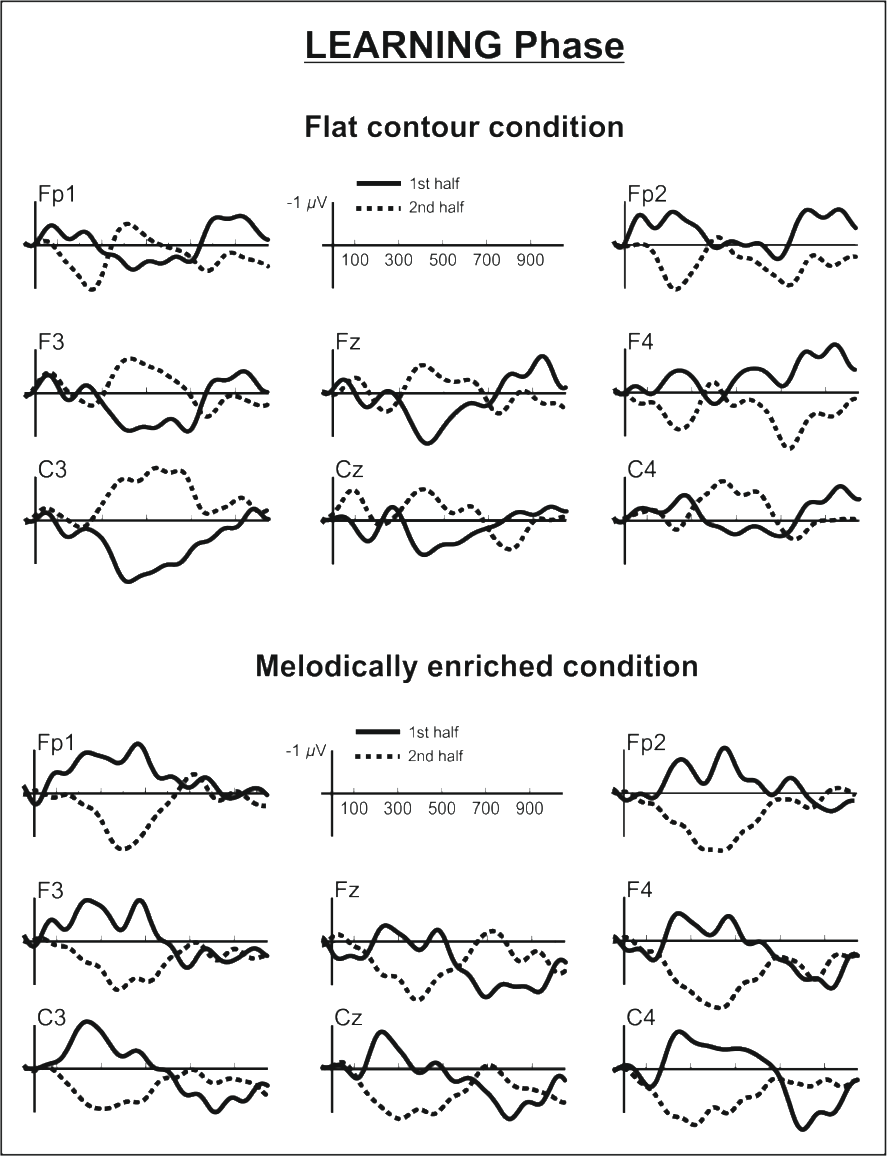
**

Supplementary Figure 1. Grand averages ERPs over 8 fronto-central electrodes across 27 newborns recorded during each block (thick = 1^st^ block, dotted = 2^nd^ block) of the learning phases in both conditions (Top: Flat contour condition; Bottom: Melodically enriched condition. Note the different ERP modulation through exposure to the streams in the two conditions. Note also a more spread ERP in the melodically enriched condition as compared to an apparent left laterlaized responses in the flat contour condition.

Supplementary figure 2


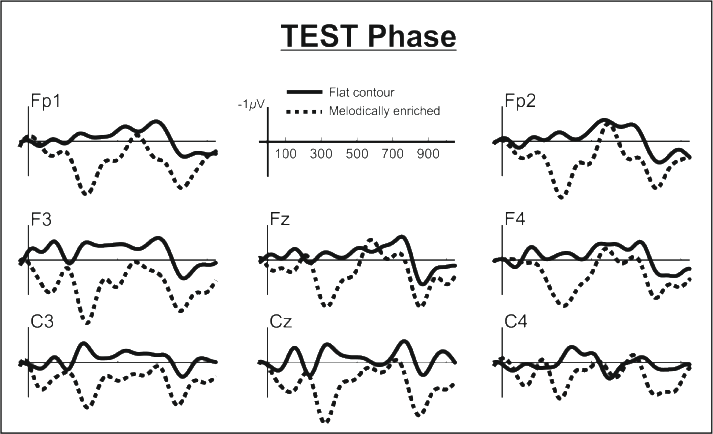


Supplementary Figure 2. Grand averages ERPs over 8 fronto-central electrodes across 26 newborns corresponding to the difference waveform (illegal minus legal) on the test phase in the flat contour (thick) and melodically enriched condition (dotted). Note the clear enhanced mismatch responses elicited by the first and last syllables of the illegal items in the melodically enriched condition as compared to the flat contour condition.
